# TransNet: Transformer-Based Point Cloud Sampling Network

**DOI:** 10.3390/s23104675

**Published:** 2023-05-11

**Authors:** Hookyung Lee, Jaeseung Jeon, Seokjin Hong, Jeesu Kim, Jinwoo Yoo

**Affiliations:** 1 Graduate School of Automotive Engineering, Kookmin University, Seoul 02707, Republic of Korea; gnrud099@gmail.com (H.L.); sing5386@naver.com (J.J.); cheongsu030536@gmail.com (S.H.); 2 Departments of Cogno-Mechatronics Engineering and Optics and Mechatronics Engineering, Pusan National University, Busan 46241, Republic of Korea; jeesukim@pusan.ac.kr; 3 Department of Automobile and IT Convergence, Kookmin University, Seoul 02707, Republic of Korea

**Keywords:** deep learning, transformer, self-attention, multi-head attention, point cloud, down sampling, classification, network

## Abstract

As interest in point cloud processing has gradually increased in the industry, point cloud sampling techniques have been researched to improve deep learning networks. As many conventional models use point clouds directly, the consideration of computational complexity has become critical for practicality. One of the representative ways to decrease computations is downsampling, which also affects the performance in terms of precision. Existing classic sampling methods have adopted a standardized way regardless of the task-model property in learning. However, this limits the improvement of the point cloud sampling network’s performance. That is, the performance of such task-agnostic methods is too low when the sampling ratio is high. Therefore, this paper proposes a novel downsampling model based on the transformer-based point cloud sampling network (TransNet) to efficiently perform downsampling tasks. The proposed TransNet utilizes self-attention and fully connected layers to extract meaningful features from input sequences and perform downsampling. By introducing attention techniques into downsampling, the proposed network can learn about the relationships between point clouds and generate a task-oriented sampling methodology. The proposed TransNet outperforms several state-of-the-art models in terms of accuracy. It has a particular advantage in generating points from sparse data when the sampling ratio is high. We expect that our approach can provide a promising solution for downsampling tasks in various point cloud applications.

## 1. Introduction

The technology for creating point clouds using 3D sensings, such as RGB-D cameras and LiDAR, is advancing rapidly, and increases in computing speeds and interest in the 3D point cloud field are drawing attention as well [1,2,3]. This has raised the importance of point clouds in various fields. Point clouds provide a detailed and accurate representation of real-world objects and environments, allowing for the precise measurements, analysis, and manipulation of 3D data. They have numerous applications in fields such as robotics, autonomous vehicles, virtual reality, architecture, and cultural heritage preservation. As the technology for creating point clouds continues to improve, we can anticipate an even greater reliance on these data structures for a wide range of applications.

Because the form of 3D point cloud data differs from that of a typical image or natural language processing (NLP) data, when point cloud research first began, new methods of point cloud generation were needed because point clouds were contained in irregular spaces with varying densities.

Initially, a method was proposed to convert 3D point cloud data into 2D images for processing. This method converts the points of 3D point cloud data into pixels of an image and treats them as images. While it has been successfully applied in the field of image processing, it does not fully reflect the complexity and diversity of 3D point cloud data. Thus, other metrics are needed to process 3D point cloud data.

Initially, projection-based [4,5] and volumetric convolution-based methods [6,7,8] were proposed to convert each point into a grid to handle a 3D point cloud and perform feature extraction using convolutional layers in the same way as conventional 2D images on the grid. Because these methods convert irregular points in 3D space into a grid format, the number of points in the grid cell is uneven, resulting in the loss of information or wasted calculations in certain cells. To overcome the problems of grid transformation, direct point-based strategies have emerged. Some methods independently model each point using multiple shared multi-layer perceptrons (MLPs) [9,10,11]. Depending on the type of convolution kernel, 3D convolution methods have emerged [12,13,14,15,16].

Point clouds are being applied to various fields, such as classification, semantic segmentation [17,18], and registration [19], instance segmentation [20,21]. These methods use point cloud data as input and aggregate local features in the last step. While they maintain accurate location information, computational costs increase linearly with the number of points, and processing high-capacity, dense 3D point cloud data remains challenging. Accordingly, a method of sampling data is proposed to reduce the amount of data in the 3D point cloud and improve processing efficiency. Previously, heuristic-based sampling methods, such as task-agnostic random sampling, fast point sampling, and grid voxel sampling, have been used. However, these methods can degrade performance because they lose information or select meaningless data from downstream tasks. Recently, a task-oriented sampling network [22,23,24] was proposed, allowing the generation of sampling that optimized the performance of downstream tasks. S-Net and SampleNet performed well for specific tasks with sampling strategies using deep learning. In addition, APSNet used the attention-based method to focus on relationships among the points. Still, these models do not fully consider the relationship information between point clouds.

**Figure 1:**
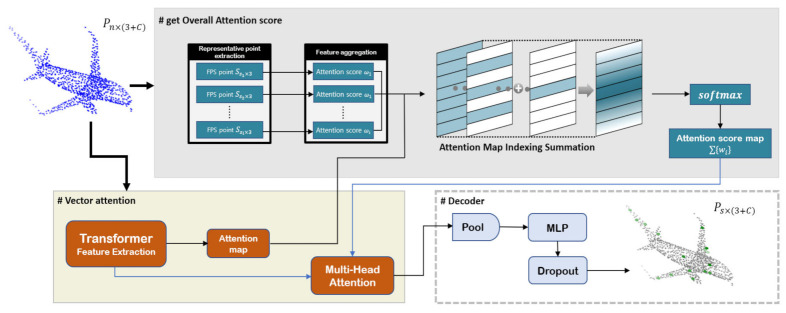
Overview of TransNet. TransNet divides the initial input into two parts. It calculates a comprehensive attention score map for each part (**top**) and vector attention (**lower left**). Then, multi-head attention is performed to understand the relationships between the points, and this process is repeated for training. In the decoder part (**lower right**), pooling and MLP are used to extract the final features. Detailed implementation methods are provided in Section 3.1 and Section 3.2.

In this paper, we propose a methodology that leverages the complete information from the input sequence to effectively interact with the task model for task-oriented sampling. TransNet is a novel transformer-based model that handles an entire sequence in parallel, capturing a long range of point cloud information and point-to-point interaction information more effectively. Feature extraction is performed by adding the embedding layer of the input and positional encoding. After generating the query, key, and value, it proceeds through the transformer [25] layer with the self-attention mechanism to effectively capture the complex interrelationships among points within the input point cloud data. By focusing on the most informative points, our method can selectively sample only the most relevant areas of the point cloud, thereby improving the efficiency of the network and the accuracy of the output. Through this approach, we can gain a more comprehensive understanding of point cloud data and easily extract meaningful features that are essential for downstream tasks (see Figure 1). Our proposed model has achieved state-of-the-art performance improvements in the field of point cloud classification. In particular, the effect is remarkable for sparse points due to the high sampling ratio. To summarize, our main contributions are threefold:

1. We propose TransNet, a novel self-attention-based point cloud sampling network, as a task-oriented objective.
2. Our approach demonstrates enhanced performance on point cloud tasks, outperforming both task-agnostic and task-oriented methods.
3. This approach effectively addresses the long-range dependency issues that are commonly encountered in point clouds. Thus, it has a notable impact on the sparsely sampled point clouds, where a high sampling ratio is required to effectively capture the underlying geometric structures.

## 2. Related Work

**Deep learning on point clouds:** Deep learning has been applied to various point cloud-related tasks, such as object detection, segmentation, and classification. For example, Qi et al. [9,10] proposed point cloud classification and segmentation and achieved state-of-the-art performance on several benchmark datasets. In addition, a deep learning model was proposed for object detection in point clouds [26,27]. Other models have been proposed for point cloud processing utilizing local features [16] and adaptive convolution operations [28]. Additionally, some studies have explored the use of generative models [29,30] for point cloud generation and reconstruction tasks. One study [31] used a local self-attention mechanism, unlike the global attention scheme used in previous studies. Furthermore, it demonstrated that vector attention methods outperformed scalar attention methods and introduced position encoding methods to properly process location information in point clouds. Although the application was different in this paper, the self-attention technique was encoded by applying the point cloud technique similar to the point transformer [31]. To preserve the location information, positional encoding was utilized, and a decoder was constructed without undergoing multiple stages.

**Point cloud sampling:** Task-agnostic algorithms, such as random sampling, uniform sampling, farthest point sampling (FPS), and grid sampling, have been widely used in the past. Among them, FPS remains a popular choice in many recent studies [31,32]. While FPS has been widely used, it may not fully consider the downstream tasks for which the sampled points are used, leading to potential performance degradation. Thus, alternative downsampling methods have recently been proposed [22,23,24]. According to Dovrat et al. [22], the efficiency and accuracy of sampling could be improved through a learnable point cloud sampling method. Lang et al. [23] introduced a novel differentiable relaxation for point cloud sampling. The authors of [24] proposed sampling attention mechanisms to enhance the relationships among points by assigning importance weights, allowing for a more effective sampling process. Our TransNet is a task-oriented sampling method that mitigates long-range dependency while viewing the relationships between points globally and locally.

**Transformer and self-attention:** Transformer and self-attention models have revolutionized machine translation and NLP [25,33]. Considering this, such methods have been increasingly used in the field of 2D image recognition [34,35]. Inspired by these findings, researchers have also attempted to apply self-attention networks to point cloud data. However, previous studies have utilized global attention on the entire point cloud, which limits their applicability to understanding large-scale 3D scenes due to high computational costs. Recently, Hengshuang et al. [31] developed a highly accurate and scalable self-attention network, specifically for large 3D scenes, using vector attention applied locally. In contrast to prior approaches, we applied a transformer locally to handle the input’s point cloud sampling, which has been shown to be highly effective.

**Nearest neighbor selection:** In recent studies, nearest neighbor (NN) methods have been widely used for information fusion. However, in the context of neural networks, the main drawback is that the selection rule is not differentiable. To address this, Goldberger et al., proposed the probabilistic relaxation of NN rules by defining categorical distributions over a set of candidate neighbors. In our study, we applied self-attention to point clouds using k NNs, allowing for a better grasp of the relationships between the points. Additionally, we incorporated skip connections to consider information from the global area.

**Positional encoding:** In the domain of deep learning models, positional encoding has been commonly utilized to encode the positional information of input data. With respect to point clouds, previous research has employed basic encoding techniques, such as Cartesian, spherical, and polar coordinates, to incorporate the position information of the points. However, these methods have limitations in terms of information loss and insufficient expressiveness. To address this issue, some studies have employed learned positional encoding techniques to incorporate more informative position information into the model. These techniques usually involve learning a continuous function to represent the position information, which can capture complex spatial relationships and patterns in point clouds.

## 3. Proposed TransNet

Here, we briefly explain the transformer and self-attention concepts. Transformer and self-attention networks are innovative and have shown impressive results in NLP [25,33] and 2D image analysis [34,35]. Recently, networks have also been applied to 3D point cloud scenes [31,32]. Self-attention can be classified into two types: dot-product attention [25] and vector attention [36]. The standard formula for dot-product attention is as follows:

(1) $$y_{i}=\underset{x_{j}\in X}{\sum }\tau \left(\alpha {\left(x_{i}\right)}^{T}\beta \left(x_{j}\right)+\delta \right)\gamma \left(x_{j}\right)$$

where $x_{i}\in X$ is a set of feature vectors, $x_{i}\;\mathrm{and}\;y_{i}$ are the input feature and output feature, respectively, α, β, and γ  are pointwise feature transformations (e.g., MLP, linear layer), and τ and δ are normalization functions (a softmax and a positional encoding function, respectively).

Unlike dot product attention, vector attention measures show similarity by calculating the distance between the input vector and the weight vector:

(2) $$y_{i}=\underset{x_{j}\in X}{\sum }\tau \left(\varepsilon \left(\mu \left(\alpha \left(x_{i}\right),\beta \left(x_{j}\right)\right)\right)+\delta \right)⊙\gamma \left(x_{j}\right)$$

where μ is a relation function (e.g., subtraction, multiplication) and ε is a mapping function (e.g., MLP) that produces attention vectors for feature aggregation.

### 3.1. Transformer-Based Sampling Layer

Traditional task-oriented sampling methods, such as S-Net [22], SampleNet [23], and APSNet [24], use PointNet models that employ convolution networks to perform feature extraction. Moreover, S-Net and SampleNet generate m points at a time, and APSNet proceeds through the sequential generation process. In this study, we introduce a novel deep-learning sampling model based on self-attention. We processed inputs by defining the query, key, and value without using the convolution network. We used vector attention and the subtraction operation between the query and key. Our vector attention process was as follows:

(3) $$y_{i}=\underset{x_{j}\in X_{knn}}{\sum }\tau \left(\varepsilon \left(\mu \left(\alpha \left(x_{i}\right),\beta \left(x_{j}\right)\right)\right)+\delta \right)⊙\gamma \left(x_{j}\right)\;$$

Moreover, taking inspiration from [31], we performed self-attention within a local neighborhood to avoid the high computational costs that arise from global self-attention.

Here, $P\in R^{n\times 3}$ denotes a point cloud that contains a given point cloud n, which is the number of point clouds. We applied feature transformation to the local region selected by KNN to generate value v. We created an attention map of the same size as the value and used the indexing sum to create an attention score map W based on the relationship between representative points (a detailed explanation is given in Section 3.2). Finally, we obtained the attention value by using multi-head attention on the attention score map W and value:

(4) $$Attention\;value=\sum_{i=1}^{n}w_{i}⊙v$$

### 3.2. Attention Score Map

**Figure 2:**
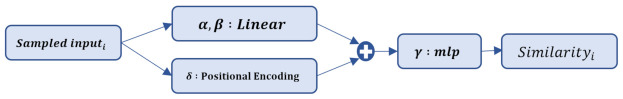
Similarity calculation. Prior to performing value v and multi-head attention, we generated attention scores for the sampled points via FPS and created an attention score map for multiple similarities. Additionally, we incorporated positional encoding to retain positional information.

We defined FPS as an algorithm for extracting representative points and performed multiple rounds of self-attention on the points extracted by FPS. With this result, we obtained the query and key for each sampled point, proceeded through a subtraction relationship, and then created a similarity in addition to Positional encoding. A description of the figure is shown in Figure 2. This process was repeated for the number of farthest point sampling performed. This resulted in the generation of an attention map that effectively encompassed all the generated values called the scatter sum. We describe the process in detail below.

First, we performed the KNN algorithm to find local features for the initial input and defined the value for local vectors. Then, we created an attention map for the empty space corresponding to the shape of the vector to calculate the distance between the input vector and the weight vector using vector attention. For the points S obtained through the FPS algorithm as S ∈P, we generated query and key vectors for the representative S points among the N points generated through the FPS algorithm and examined their similarity through the subtraction relationship of the two generated vectors, as described in Equation (5).

(5) $$w_{i}=\underset{x_{j}\in X_{knn}}{\sum }\alpha \left(x_{i}\right)-\beta \left(x_{j}\right)$$

**Figure 3:**
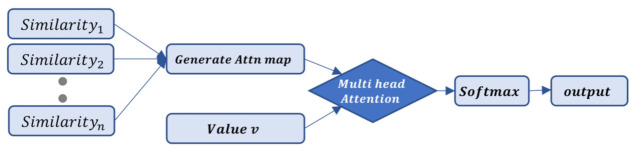
Transformer sampling layer.

We added this to the index corresponding to the attention score map W. We repeated this process for all set ratios R and applied a normalization activation function to the resulting attention score map:

(6) $$W=idx(w_{1})+idx\left(w_{2}\right)+\ldots +idx\left(w_{i}\right)$$

followed by performing multi-head attention with the initially obtained value v. Details are described in Figure 3.

To summarize the process before entering the Decoder, the initial point cloud was divided into two steps. In the first step (yellow area in Figure 1), the value v of the input was obtained, and an empty attention map of the same size was created. In the second step (gray area in Figure 1), a representative point was drawn through the FPS, and the query and key were obtained for each of the selected $S_{s_{i}\times 3}$; the similarity was obtained through this. Subsequently, we added weights to each index of the attention map generated in the first step to create a single attention map, which was then used to perform vector attention with the value v.

By identifying the interrelationships between the points in the input sequence, self-attention facilitated a better understanding of the relationships between each point, resulting in superior performance in handling long-term dependencies. Compared to traditional models that use LSTM [37], our proposed model enables the parallel processing of input sequences, resulting in superior performance, particularly in scenarios with high sampling ratios.

### 3.3. Decoder

In the decoding stage, a max-pooling operation was conducted to collect the extracted features. To generate the final output, a fully connected network (FCN) was utilized. To mitigate the loss of positional information that was caused by employing a linear MLP and positional encoding was further incorporated. Additionally, dropout was employed as a regularization technique to prevent overfitting. The result contained a number of s points for the task. With this result, we performed multi-task learning. By adopting a multi-task learning approach, all tasks could be efficiently processed within a single model. The concurrent training of these two models enabled us to effectively leverage the shared data distribution and consider more inter-task correlations, thus further improving the model’s performance. Further details regarding the model’s architecture are illustrated in Figure 1 (lower right).

### 3.4. Loss

We applied supervised learning and used two types of loss: task loss $L_{\mathrm{task}}$ and sampling loss $L_{\mathrm{sample},}$ to train TransNet, where Total loss is the sum of the weights added to these two losses. The sampling loss $L_{sample}$ aimed to minimize the distance between the points sampled from S, and the corresponding points in P, while also ensuring that the sampled points were spread out as much as possible across the original point cloud P.

$L_{task}$ can be defined as the cross-entropy loss for classification. Additionally, the formula for this is as follows:

(7) $$L\left(\hat{y},y\right)=-\overset{C}{\underset{i=1}{\sum }}y_{i}\log\left({\hat{y}}_{i}\right)\;$$

$L_{sample}$ is the sum of two things: average neighbor loss and maximum neighbor loss. Given two-point sets $Q_{1}$ and $Q_{2}$, the average nearest neighbor loss can be denoted as:

(8) $$L_{a}\left(Q_{1},Q_{2}\right)=\frac{1}{\left|Q_{1}\right|}\underset{q_{1}\in Q_{1}}{\sum }\underset{q_{2}\in Q_{2}}{\min}{\left|\left|q_{1}-q_{2}\right|\right|}_{2}^{2}$$

Additionally, maximal nearest neighbor loss can be given as:

(9) $$L_{m}\left(Q_{1},Q_{2}\right)=\underset{q_{1}\in Q_{1}}{\max}\underset{q_{2}\in Q_{2}}{\min}{\left|\left|q_{1}-q_{2}\right|\right|}_{2}$$

The sampling loss is then given by:

(10) $$L_{sample}\left(Q,P\right)=L_{a}\left(Q,P\right)+\beta L_{m}\left(Q,P\right)+\left(\gamma +\delta \left|Q\right|\right)L_{a}\left(P,Q\right)$$

where β,γ and δ are the hyperparameter that adjusts the size between losses. In conclusion, the total loss is then as follows:

(11) $$L_{total}=L_{task}+\lambda L_{sample}\left(Q,P\right)$$

where *λ* is a hyperparameter value that adjusts the value between the task loss and the sample loss.

## 4. Experimental Results and Discussion

In this section, we demonstrate that the performance of our TransNet is superior to that of existing sampling methods in various fields. We conducted experiments in the classification and registration domains and proved that the performance was particularly good in areas with high point cloud sampling ratios. We experimented with two variations of TransNet (W.O indexing summation) and TransNet. The former is a model that applies the transformer architecture. This is a method of applying the Transformer method by generating queries, keys, and values based on existing information without using FPS points. The latter is a model that incorporates an attention map into the transformer architecture. This last model performed better, and here we experimented by comparing the latter model with those of other papers.

We implemented TransNet in PyTorch, setting the batch size, SGD optimizer with momentum, and weight decay to 128, 0.9, and 0.0001, respectively. In addition, we conducted 400 epochs of training in all the experiments. For classification, we used the ModelNet 40 dataset [38]. We performed experiments on 1024 points that were uniformly sampled. To train and evaluate our models, we used the train-test split dataset provided on the official website. We used instance-wise accuracy as a metric to evaluate the classification results of each sample in multi-class classification problems. Each sample belonged to a single class, and if the predicted class by the classification model matched the actual class, the sample was considered “correctly classified”. Therefore, instance-wise accuracy represents the proportion of samples that were correctly classified among all the samples. Furthermore, we focused our experiments on sparse points with sampling ratios of 16, 32, 64, and 128, which had previously shown an inferior performance in all papers. In this study, we conducted an experimental analysis using PointNet, which led us to assume the outcomes of Table 1.

The sampled experiment would not surpass the accuracy achieved by the original PointNet prior to sampling. Therefore, we contended that using other state-of-the-art models can also lead to higher sampling accuracy. For example, in the case of classification, since PointNet was defined as the underlying model, it was assumed that the performance of the unsampled PointNet would not be exceeded by any sampled model.

**Figure 4:**
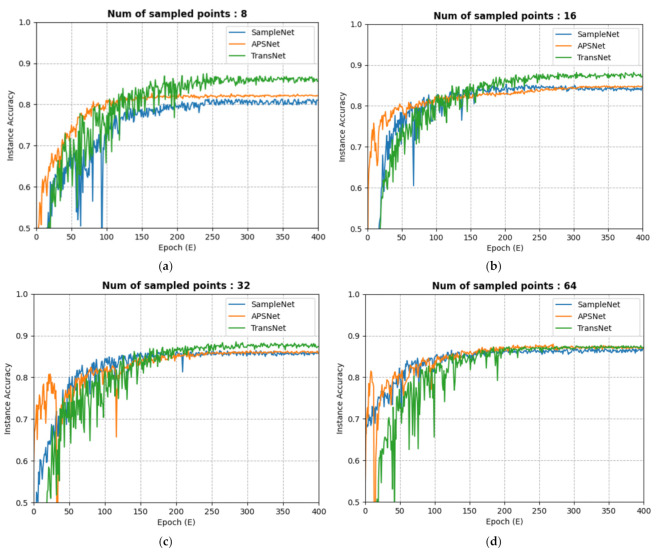
Instance accuracy. This is the result of training three sampling models (SampleNet, APSNet, TransNet) on the ModelNet40 dataset after uniformly sampling 1024 examples. The sampling ratios used were 128, 64, 32, and 16. For example, if 8 examples were sampled from 1024, the sampling ratio would be 128. TransNet achieved much better performance in sparse areas such as (**a**,**b**) and showed superior performance in other areas. Less sparse (**c**,**d**) can also see similar or higher levels of results than existing papers. The classification accuracies are shown in Table 1.

**Figure 5:**
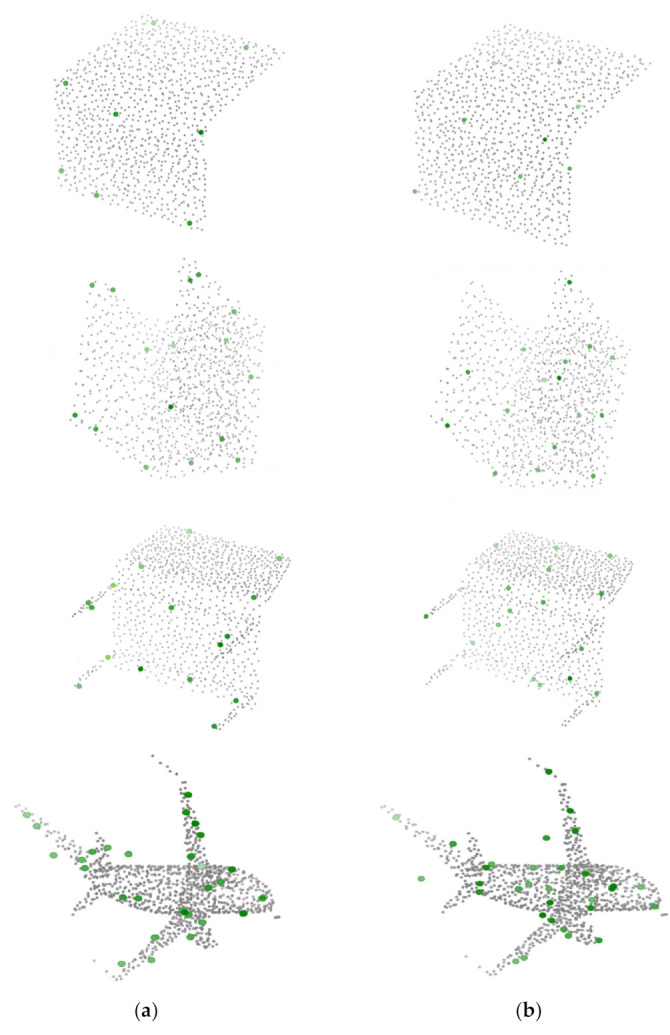
Visualized Sampled Points. Results for 8, 16, and 32 sampling points (green) on ModelNet40. The results on the left are from TransNet (**a**), and those on the right are from APSNet (**b**). Moreover, from top to bottom, the objects are a laptop, nightstand, chair, and airplane. The gray points represent the original ground truth, and the green points were generated. From the overall shape, the result value of our TransNet (**a**) szx more evenly expressed than the result value of APSNet (**b**). Overall, for square objects (laptop, desk), the method tended to have a better grasp of the ends, while for objects such as chairs, it tended to have a better grasp of the legs. Detailed class-specific results are shown in Table 2.

As Figure 4 shows, we have created a model that exceeds the performance of existing models for the experimental results of sampling 8, 16, 32, 64 for 1024 points each. In particular, we demonstrated that the sparse points (the results of sampling 8 or 16) resulted in greater deviations from other models and that our model was more robust in sparse data. In Figure 5, we present a sampling comparison experiment of our model and comparison model on the same object, from which we can clearly see the difference. Our model samples objected much more evenly and reasonably than an APSNet model’s sampling result, demonstrating its superior performance in classification results. As a result, our TransNet had a better grasp of corners and characteristic parts than existing methods. However, all deep learning models exhibited a tendency to mislead in certain areas (table legs, flower in a pot, etc.), with many weak characteristics in common, and this problem remains to be solved.

**Table 2:** Class-specific accuracies of two sampling methods on ModelNet40. All experiments were conducted in the same environment.

Sampling Ratio		Laptop	Chair	Nightstand	Airplane
128	SampleNet	81.32	91.38	38.18	97.51
	APSNet	83.33	95.56	42.18	98.52
	TransNet	**85.32**	**96.49**	**51.88**	**99.1**
64	SampleNet	85.23	94.38	59.38	98.11
	APSNet	88.88	95.52	64.67	99.49
	TransNet	**91.42**	**96.52**	**65.92**	**99.55**
32	SampleNet	89.29	86.38	67.21	98.89
	APSNet	90.90	94.68	67.39	**99.50**
	TransNet	**90.91**	**97.05**	**72.50**	99.00

## 5. Conclusions

Transformer algorithms have been expanded beyond the natural language process into various fields. We applied this algorithm to the field of point cloud sampling and achieved successful results. In this paper, we proposed a novel transformer-based point cloud sampling network to achieve precision performance. While S-Net and SampleNet generated the sampling process using MLP-based methods, and APSNet used an attention-based model, TransNet employed a multi-head self-attention technique in the down-sampling process. In addition, assuming that FPS is a representative point for viewing general-purpose information, we created an attention map that collected information after proceeding through several FPS algorithms and indexed it for each location while applying multi-head attention. This strategy improved the relationship between each point in the learning procedure while it also learned simultaneously with task models and reasonably understood relationships alongside alleviating long-range dependencies. The proposed TransNet demonstrated a better performance in terms of precision, especially on sparse data. Moreover, the proposed sampling method could be applied to various kinds of point cloud deep learning networks. Thus, its usefulness would be meaningful in many practical scenarios.

## 6. Ablation Study

### 6.1. K-Nearest

After extracting the representative points with FPS, we applied the K-nearest neighbor algorithm to find the neighbor points for the representative points and identify the association between the points. The neighborhood size k is the number of neighbors in the representative point P of a point q ∈ Q. We evaluated the impact of the hyperparameter, k, by training multiple progressive TransNet for classification with different values of k. TransNet was applied as k = 16, and experiments were conducted on k ∈ {4, 8, 16, 32}, respectively. The results of the experiment are shown in Table 3. If the neighbor was smaller (k = 4 or k = 8), there might not have been enough context for the model to make predictions. If the neighbor was larger (k = 32), each self-attention layer had a large number of data points, many of which could be further away and less relevant. This could result in excessive noise during processing with the potential to degrade the accuracy of the model.

### 6.2. Additional Experiments

As mentioned briefly earlier, when applying the Task model without the sampling model, higher accuracy was achieved compared to the application of the sampling model. When tested with PointNet, an accuracy of 90.08 was obtained. To be as close to this target as possible, we made several attempts, such as adding dropout, skipping connections, or experimenting with added positional encoding to add positional information in different parts of the model. The activation function also conducted many experiments, such as ReLu, Leaky ReLu, and ELU. As a result, we set the probability of the dropout to 0.1 and added positional encoding to the encoder and decoder portions of the model, respectively. The activation function performed best when using ReLu.

## Figures and Tables

**Table 1:** Classification accuracies of five sampling methods on ModelNet40. All experiments were conducted in the same environment.

Sampling Ratio	128	64	32	16
RS	8.7	24.87	54.53	79.26
FPS	24.3l	55.12	76.92	87.53
SampleNet [23]	80.71	85.32	86.38	87.10
APSNet [24]	82.72	84.89	86.66	**88.00**
TransNet	**87.47**	**88.16**	**88.49**	87.88

**Table 3:** The accuracy of TransNet according to K. All experiments were conducted in the same environment, and we adopted K = 16.

K-Size	TransNet-4			TransNet-8			TransNet-16			TransNet-32		
Sampling ratio	128	64	32	128	64	32	128	64	32	128	64	32
Instance Accuracy	85.36	86.24	85.32	85.95	86.68	85.93	**87.47**	**88.16**	**88.49**	86.21	87.67	87.58

## Data Availability

Not applicable.
